# Resistance to doxorubicin‐induced proteinuria and proteolytic activation of ENaC in 129S2/SvPas mice

**DOI:** 10.14814/phy2.70667

**Published:** 2025-12-08

**Authors:** Evan C. Ray, Ivy Liu, Niloofar Momenzadeh, Kennedy Szekely, Allison L. Marciszyn, Tracey Lam, Andrew J. Nickerson, Thomas R. Kleyman

**Affiliations:** ^1^ Department of Medicine University of Pittsburgh Pittsburgh Pennsylvania USA; ^2^ Department of Cell Biology University of Pittsburgh Pittsburgh Pennsylvania USA; ^3^ Department of Pharmacology and Chemical Biology University of Pittsburgh Pittsburgh Pennsylvania USA

**Keywords:** 129sv, adriamycin, albuminuria, doxorubicin, ENaC, Prkdc, proteinuria

## Abstract

Doxorubicin treatment of mice represents a convenient model to study the effects of proteinuria on proteolytic processing of the epithelial Na^+^ channel (ENaC) and urinary Na^+^ and fluid handling. Prior studies have shown enhanced ENaC γ subunit proteolysis and Na^+^ and fluid retention in 129S1/SvImJ mice treated with doxorubicin. We examined whether 129S2/SvPas mice could be used to study doxorubicin‐induced proteinuria. 129S2/SvPas mice treated with 18 μg/g doxorubicin exhibited significantly reduced urinary albumin/creatinine compared to that described for 129S1/SvImJ mice. Proteolytic processing of ENaC's γ subunit and sensitivity of urinary Na^+^ and K^+^ to the ENaC blocker, benzamil, were not enhanced by doxorubicin. Differences in the DNA repair enzyme, DNA‐activated protein kinase (DNA‐PKcs) may contribute to the difference in doxorubicin susceptibility of 129S2/SvPas and 129S1/SvImJ mice. Sequencing of the *Prkdc*, encoding DNA‐PKcs, showed that 129S2/SvPas mice lack a polymorphism (Arg2140Cys) that has previously been implicated in doxorubicin sensitivity in 129S1/SvImJ mice. The status of this polymorphism in various experimental mouse strains, and therefore implications for studying doxorubicin‐induced proteinuria, is discussed.

## 1 INTRODUCTION

The epithelial sodium channel (ENaC) represents the rate‐limiting step for Na^+^ reabsorption in the distal nephron. This channel is classically considered to be composed of three subunits—a homologous α, β, and γ subunit (Kashlan et al., 2024; Sheng et al., 2013). Proteolytic processing of the channel plays a key role in channel activity. The channel's α subunit contains two furin cleavage sites that are usually cleaved as the subunit passes through the cell's *trans*‐Golgi apparatus (Carattino et al., 2006). Between these two sites lies an inhibitory tract that suppresses channel open probability (P_O_). Cleavage of both furin sites allows dissociation of the inhibitory tract, enhancing channel activity and promoting tubular Na^+^ reabsorption (Nickerson et al., 2024). Similarly, the γ subunit harbors an inhibitory tract that is flanked proximally by a furin cleavage site and distally by a series of sites that can be cleaved by an assortment of extracellular proteases (Anand et al., 2022; Ray et al., 2015) (Carattino et al., 2008; Kashlan et al., 2024). Removal of the γ subunit's inhibitory tract dramatically enhances channel activity, increasing P_O_ to near unity, regardless of the cleavage state of the channel's α subunit (Carattino et al., 2008).

Among the proteases capable of cleaving the γ subunit at sites distal to its inhibitory tract is plasmin (Passero et al., 2008; Svenningsen et al., 2009). Plasmin has been detected in the urine in the context of glomerular disease and proteinuria in many settings. In humans, this includes individuals with diabetic nephropathy, severe hypertension, and pre‐eclampsia (Andersen et al., 2015; Buhl et al., 2012, 2014; Ray et al., 2018; Unruh et al., 2017). That glomerular disease results in urinary plasmin excretion is demonstrated by laboratory models, including diabetic or puromycin aminonucleoside (PAN)‐treated rats (Andersen et al., 2015; Passero et al., 2008; Staehr et al., 2015; Svenningsen et al., 2009; Yu et al., 2005), 129S1/SvImJ mice treated with doxorubicin (Bohnert et al., 2018; Bohnert & Artunc, 2018; Haerteis et al., 2018), and C57Bl/6 mice with glomeruli deficient in podocin (Xiao et al., 2021).

Evidence for enhanced cleavage of ENaC's γ subunit has also been found in proteinuric kidney disease, including humans with proteinuria (Zachar et al., 2015), PAN‐treated rats (Lourdel et al., 2005), and doxorubicin‐treated mice in the 129S1/SvlmJ background (Artunc et al., 2022; Bohnert et al., 2019; Bohnert, Essigke, et al., 2021; Haerteis et al., 2018). This observation has led to the hypothesis that activation of ENaC through the action of blood‐stream proteases (such as plasmin) that are leaked into the nephron through damaged glomeruli could contribute to the urinary Na^+^ retention, volume overload, and hypertension that accompany proteinuric kidney disease (Andersen et al., 2015; Hamm et al., 2010; Passero et al., 2010; Ray et al., 2015; Svenningsen et al., 2011, 2012, 2013, 2015). Indeed, several authors have suggested that ENaC blocking medications should be considered in the treatment of proteinuria‐associated hypertension (Oxlund et al., 2014; Schork et al., 2024; Staehr et al., 2015; Unruh et al., 2017).

Despite these associations, a causal relationship between ENaC activation and proteinuria‐associated hypertension remains to be conclusively established, and a laboratory model including both hypertension and proteinuria‐associated enhancement of ENaC cleavage remains desirable. Mice in the 129/Sv background seem to be an appealing model. These mice exhibit hypertension more readily than C57Bl/6 mice (Hartner et al., 2003; Waeckel et al., 2012; Yang et al., 2005). A substrain of 129/Sv mice, those in the 129S2/SvPas background, are susceptible to hypertension in response to dietary electrolyte manipulation alone (Pitzer et al., 2022; Pitzer Mutchler et al., 2023). Another 129/Sv substrain, those in the 129S1/SvImJ background, exhibit significant proteinuria in response to a single doxorubicin injection (Bohnert et al., 2018; Bohnert & Artunc, 2018; Bohnert, Essigke, et al., 2021; Haerteis et al., 2018). These mice demonstrate significantly more proteinuria in response to doxorubicin than do mice in the C57Bl/6 strain, an observation that has been attributed to a polymorphism reducing expression of the *Prkdc* gene, encoding the catalytic subunit of DNA‐dependent protein kinase (DNA‐PKcs), involved in DNA double‐strand break repair (Papeta et al., 2010; Watanabe et al., 2021).

We previously demonstrated that mice in the 129S2/SvPas background with a mutation in ENaC's γ subunit preventing proteolytic processing by furin exhibit only a mild phenotype including subtle impairment in fluid volume retention on a low sodium diet, and mild reduction in flow‐mediated K++ secretion in the collecting duct (Ray et al., 2024). We hypothesized that mice in the 129S2/SvPas background could be used to examine the effects of proteinuria on fluid volume retention using a well‐established model of proteinuria in mice: doxorubicin treatment. We observe that doxorubicin induces significantly less urinary protein than that described in published reports, likely because of genetic differences in 129S2/SvPas mice and mice used in previously described experiments (129S1/SvImJ and Balb‐C mice) (Bohnert, Gonzalez‐Menendez, et al., 2021; Faiola et al., 2003; Papeta et al., 2010; Watanabe et al., 2023). The small quantity of proteinuria observed in 129S2/SvPas mice in our experiments was not sufficient to enhance proteolytic processing of ENaC, stimulate body fluid retention, change blood K^+^ or total CO_2_ (tCO_2_), or change urinary Na^+^ or K^+^ excretion in response to the ENaC blocker, benzamil. These findings show that mice in the 129S2/SvPas background may not be suitable for studies examining the relationship between proteinuria and proteolytic processing of ENaC in the kidney.

## 2 MATERIALS AND METHODS

### 2.1 Animal care

Male and female mice were in the 129S2/SvPas background (Charles River, strain 287). Mice were housed at the University of Pittsburgh Department of Laboratory Animal Research on a 12‐h light: dark cycle. Mice received standard mouse chow (Prolab Isopro RMH 3000) and were sacrificed at 11–20 weeks of age under isoflurane anesthesia. Experiments were approved by the University of Pittsburgh Institutional Animal Care and Use Committee.

### 2.2 Doxorubicin administration

Doxorubicin HCl (Fisher Scientific, CAS No. 25316‐40‐9, catalog No. AAJ64000MA) was dissolved in 70% EtOH, then diluted to 5 mg/mL in sterile 0.9% NaCl solution (final ethanol concentration: 3.5%). Dissolved doxorubicin was administered retro‐orbitally to mice under isoflurane anesthesia at a dose of 18 mg/kg body weight, using a 30‐g needle and an infusion pump (New Era Pump Systems, Inc.) with an infusion rate of 10 μL/second.

### 2.3 Urine collection and analysis

Spot urine specimens before and 10 days after doxorubicin injection were collected between 7 and 8 am. (The same mice were compared before vs. after treatment.) For urine collection after benzamil administration, benzamil (Fisher Scientific, CAS No. 161804‐20‐2, catalog No. 33‐805‐0) was dissolved in sterile saline +0.1% DMSO and administered intraperitoneally to (doxorubicin‐treated vs. untreated) mice at a dose of 1.5 mg/kg body weight (Nickerson et al., 2023). Urines were then collected for 5 h in mini‐metabolic cages starting at approximately 1:00 pm. Urine electrolytes were measured using an EasyLyte Electrolyte Analyzer (Medica Corporation). Albumin was measured using a mouse ELISA kit (Bethyl Laboratories, Fischer Scientific catalog No. NC0096469), after diluting specimens 1:45,698. Creatinine was measured using an Enzymatic Creatinine Reagent Set (Pointe Scientific, catalog No. NC1274849).

### 2.4 Blood metabolite measurement

Blood was collected from mice anesthetized under isoflurane via cardiac ventricular puncture with a heparin‐pretreated syringe. Whole‐blood metabolites were measured using an iSTAT hand‐held analyzer (Abbot).

### 2.5 Immunoblot

Whole kidneys were lysed in CelLytic MG lysis buffer (Sigma #C3228) at 1:20 weight/volume, then centrifuged at 20,000 rcf for 20 min. Supernatants were removed and 20 μg of protein was digested with PNGase F (New England Biolabs #P0704) to deglycosylate proteins. Lysates were then subjected to SDS‐PAGE and immunoblot using an antibody directed against the ENaC γ subunit's C‐terminus (StressMarq, #SPC‐405) as previously described (Ray et al., 2024). Membranes were incubated in primary antibody diluted 1:1000 in tris‐buffered saline with 0.1% Tween‐20 (TBST) + 5% milk at 4°C overnight, with agitation. The following day, membranes were washed 4 times in TBST, then incubated in HRP‐conjugated goat anti‐rabbit secondary antibody (Thermo Scientific, #31460), diluted 1:10,000 in TBST +5% milk for 2 h at room temperature. After washing again in TBST, membranes were developed in Clarity Max substrate solution (Biorad) and imaged using a Chemidoc imaging system (Biorad). Band densitometry was performed using ImageJ.

### 2.6 DNA sequencing

PCR amplification of *Prkdc* was performed with forward primer GCCATGATCCTTAGCAAGTG and reverse primer GCCTAAGGTAAGGTAAGGTGCTGTA (Yu et al., 2001). Sanger sequencing was performed by Genewiz.

## 3 RESULTS

To examine the association of proteinuria and ENaC γ subunit cleavage, 129S2/SvPas mice were treated with doxorubicin. Previous studies suggested that a dose of 14.5 μg/g of doxorubicin was sufficient to induce proteinuria with a minimum of mortality in 129S1/SvImJ mice (Bohnert et al., 2018; Bohnert & Artunc, 2018; Haerteis et al., 2018). Preliminary dose–response testing suggested that 14.5 μg/g of doxorubicin in 129S2/SvPas mice induced only minimal proteinuria, but 20 μg/g caused lethality in some mice. Therefore, we used a dose of 18 μg/g in our experiments. No mice injected with this dose of doxorubicin died, but the degree of proteinuria observed was mild compared to that previously observed in the literature, as discussed below.

**FIGURE 1:**
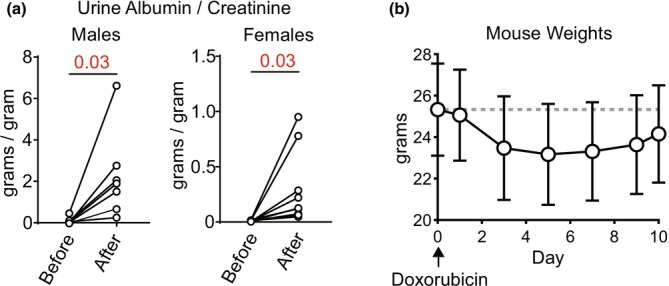
Doxorubicin induces proteinuria in 129S2/SvPasCrl males more effectively than females. Spot urines were collected from mice, then mice were injected retro‐orbitally with doxorubicin at 18 μg/g body weight. After 10 days, spot urine specimen samples were again collected. (a) Both male and female mice exhibited a significant increase in proteinuria following doxorubicin. (*N* = 7 males, 10 females. Numbers in pair‐wise comparisons represent *p* values, as determined using Student's *t*‐test). (b) Male mice exhibited a decline in body weight following injection with doxorubicin. (*N* = 12 mice. Error bars represent SD).

In prior studies, urinary protein in response to doxorubicin often peaked at 7 to 10 or more days (Bohnert & Artunc, 2018; Bohnert, Gonzalez‐Menendez, et al., 2021; Wang et al., 2000). We therefore performed analysis before doxorubicin and again 10 days after injection. Urinary albumin (or protein in general) correlates with urinary excretion of proteases capable of activating ENaC, such as plasmin (Andersen et al., 2015; Buhl et al., 2012; Oxlund et al., 2014; Ray et al., 2018; Staehr et al., 2015; Unruh et al., 2017). Urine with plasmin is enzymatically capable of activating ENaC in vitro (Buhl et al., 2012, 2014; Svenningsen et al., 2009). The single injection of doxorubicin at 18 μg/g induces albuminuria at 10 days in most mice (Figure 1a), but produces significantly more albuminuria in males (16.4 +/− 10.0 g albumin/g creatinine, compared with 1.9 +/− 2.5 g/g before doxorubicin) than in females (1.0 +/− 1.0 g/g, compared with 0.2 +/− 0.2 g/g). Further studies therefore focus on male mice. Of note, even the male mice exhibit an order of magnitude less albuminuria than has been described in 129S1/SvImJ mice (roughly 150 grams albumin/g creatinine 10 days after injection with 14.5 μg/g of doxorubicin) (Bohnert, Essigke, et al., 2021).

Doxorubicin causes weight loss soon after injection, but in some studies this initial weight loss reverses, leading to an increase in body weight beyond the starting body weight and peaking around day 10 (Bohnert et al., 2019; Bohnert, Essigke, et al., 2021; Bohnert, Gonzalez‐Menendez, et al., 2021). This increase in body weight is presumably fluid‐volume mediated. In 129S2/SvPas, the expected early decrease in body weight occurs, consistent with general illness related to doxorubicin injection, but this weight loss is not followed by an increase in body weight, suggesting a lack of fluid volume retention (Figure 1b).

**FIGURE 2:**
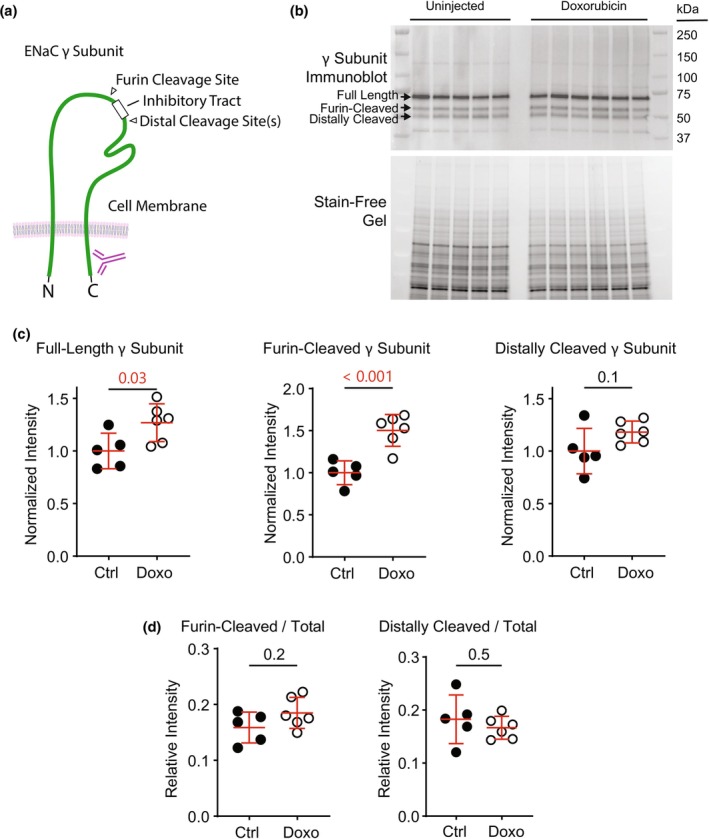
Doxorubicin injection in 129S2/SvPasCrl males does not enhance proteolytic cleavage of ENaC's γ subunit. (a) A schematic is shown depicting cleavage sites in ENaC's γ subunit and the antibody used for immunoblot. (b) Immunoblot of ENaC's γ subunit from deglycosylated whole kidney lysates. Each lane represents a kidney lysate from one mouse. (c) Quantification of full‐length γ subunit or cleavage products (normalized to stain‐free gel protein intensity). Full‐length γ subunit and furin‐cleaved C‐terminal fragment abundances increase in doxorubicin‐treated mice compared to untreated controls. Distally cleaved γ subunit protein abundance does not increase significantly. (d) Abundance of the furin‐cleaved or distally cleaved proteolytic fragments relative to the total (full‐length + furin‐cleaved + distally cleaved) γ subunit abundance is not enhanced by treatment with doxorubicin. (Ctrl, control mice untreated with doxorubicin; Doxo, doxorubicin‐treated mice. Numbers in pair‐wise comparisons represent *p* values, as determined using Student's *t*‐test.)

We next examined the abundance of ENaC's γ subunit and its cleavage products in whole kidney lysates from untreated control mice or mice injected with doxorubicin (Figure 2a,b). The abundance of full‐length ENaC γ subunit (72 kDa) increases in mice treated with doxorubicin (Figure 2c). This observation may be attributable to increased aldosterone (Yang et al., 2020). Doxorubicin treatment raises aldosterone levels in mice (Bohnert et al., 2018, 2019; Bohnert, Essigke, et al., 2021). Increased aldosterone may result from reduced oral intake, transposition of intravascular fluid to the interstitial space, or reduced cardiac output, each of which is a well‐established adverse effect of doxorubicin therapy (Cella et al., 2024; Kong et al., 2022; Sheibani et al., 2022).

In immunoblots of the γ subunit, a 57 kDa band represents the γ subunit cleaved at its furin consensus site, proximal to the subunit's inhibitory tract (Ray et al., 2024). Overall abundance of this cleavage product also increases in mice treated with doxorubicin. A smaller, 52 kDa, band represents the γ subunit that has been cleaved distal to its inhibitory tract. This region of the subunit includes a cleavage site for plasmin, which is leaked into the urine in the context of proteinuric kidney disease (Passero et al., 2008; Ray et al., 2018; Svenningsen et al., 2009; Wu & Hoak, 1973). We therefore expected to see an increase in the abundance of the 52 kDa band in doxorubicin‐treated animals. However, the abundance of the distal cleavage product is not significantly elevated by doxorubicin treatment (Figure 2c). This finding is strikingly different than previously described in 129S1/SvImJ mice, where doxorubicin treatment induced a more than 2.5‐fold increase in the abundance of the distal cleavage product in response to doxorubicin (Bohnert, Essigke, et al., 2021). In the present study, the relative abundances of γ subunit cleavage products relative to total γ subunit protein provided no indication that treatment with doxorubicin enhanced proteolytic cleavage of the subunit (Figure 2d).

**FIGURE 3:**
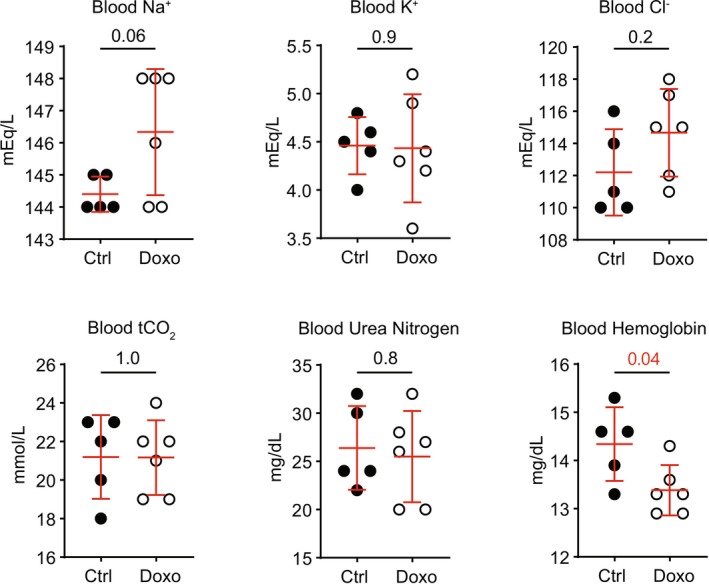
Blood metabolites in male 129S2/SvPasCrl mice 10 days following doxorubicin treatment are shown (Ctrl, control mice untreated with doxorubicin; Doxo, doxorubicin‐treated mice. (Numbers in pair‐wise comparisons represent *p* values, as determined using Student's *t*‐test).

To further examine mice for evidence of ENaC activation, plasma electrolytes were examined (Figure 3). Mice exhibit no differences in whole‐blood Na^+^, K^+^, Cl^+^, tCO_2_, or urea nitrogen (BUN). However, blood hemoglobin levels are lower, as previously reported (Artunc et al., 2008).

**FIGURE 4:**
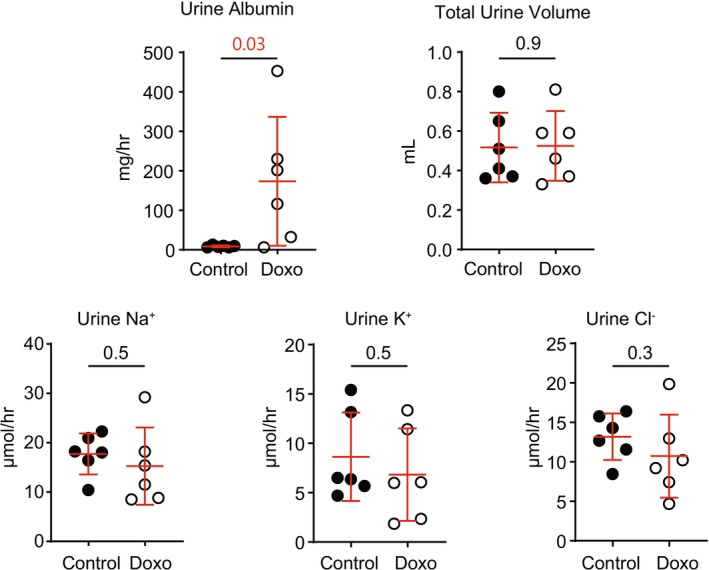
In male 129S2/SvPasCrl mice, injection with doxorubicin does not alter benzamil‐induced urinary electrolyte excretion. Ten days after treatment with doxorubicin, treated or untreated control mice were injected intraperitoneally with 1.5 mg/kg of benzamil and urine was collected over 5 h. Urine albumin was significantly increased in doxorubicin‐treated animals, but urine volume and excretion of Na^+^, K^+^, and Cl^−^ were not different between groups. (Doxo, doxorubicin‐treated mice. Numbers in pair‐wise comparisons represent *p* values, as determined using Student's *t*‐test).

Next, doxorubicin‐treated mice were evaluated for altered electrolyte excretion in response to a pharmacologic blocker of ENaC, benzamil (Figure 4). Increased benzamil‐associated natriuresis or decreased kaliuresis would be consistent with activation of ENaC. Doxorubicin‐treated animals again exhibit increased urinary albumin excretion, as in the previous experiment, but exhibit no difference in urine volume or excretion of Na^+^, K^+^, or Cl^−^ following benzamil administration.

To understand the apparent difference in doxorubicin sensitivity of 129S2/SvPas and 129S1/SvImJ mice, we sequenced a portion of *Prkdc*, encoding the catalytic subunit of DNA‐activated protein kinase (DNA‐PKcs) that participates in DNA double‐strand break repair. Polymorphism rs4164948 in *Prkdc* has been implicated in enhanced sensitivity of 129S1/SvImJ and Balb‐C mice to doxorubicin (Bohnert, Gonzalez‐Menendez, et al., 2021; Faiola et al., 2003; Papeta et al., 2010; Watanabe et al., 2023). This polymorphism results in a substitution of arginine at position 2140 with cysteine (Arg2140Cys), reducing DNA‐PKcs expression and increasing the sensitivity of mice to mutagens (Bohnert, Gonzalez‐Menendez, et al., 2021; Faiola et al., 2003; Papeta et al., 2010; Watanabe et al., 2021; Yu et al., 2001). Proteinuria occurring in response to doxorubicin treatment is suggested to occur because of podocyte DNA damage and cell death (Papeta et al., 2010). We sequenced the *Prkdc* locus in four individual 129S2/SvPas mice and found that they do not harbor the Arg2140Cys substitution, making them more similar to C57Bl/6 than 129S1/SvImJ mice in this regard. This genotypic difference likely contributes to decreased sensitivity of 129S2/SvPas mice to doxorubicin‐induced proteinuria, compared with 129S1/SvImJ mice.

## 4 DISCUSSION

129S2/SvPas mice treated with 18 μg/g doxorubicin exhibit only mild albuminuria and fail to exhibit signs of enhanced proteolytic activation of ENaC, including an increase in body weight consistent with fluid volume retention, enhanced cleavage of ENaC's γ subunit, altered blood K^+^ or tCO_2_, and altered natriuresis or kaliuresis in response to benzamil. These findings differ from previous reports demonstrating enhancement in ENaC proteolysis and fluid retention in doxorubicin‐treated mice in the 129S1/SvImJ background. The likely explanation for the failure to enhance proteolytic processing of ENaC in the present study is the significant reduction in proteinuria as compared with previously published studies in 129S1/SvImJ mice. However, enhanced ENaC γ subunit cleavage has been observed in some models with comparable levels of proteinuria (Table 1). Thus, the degree of proteinuria may not be the sole determinant of ENaC γ subunit proteolytic processing.

**TABLE 1:** Comparison of proteinuria and ENaC γ subunit cleavage in various mouse models.

Mouse background	Sex	Proteinuria model	Kidney tissue	Fold‐increase in proteinuria	Fold‐increase in distally cleaved/full‐length γ subunit	References
129S2/svPas	Male	Doxorubicin	Whole	8.6^a^	0.93	Present study
129S1/SvIMJ	Both^b^	Doxorubicin	Cortex	>100^c^	2.60	Bohnert, Essigke, et al. (2021)
C57Bl/6^d^	Female	Auto‐immune (anti‐mouse glomerular basement membrane serum)	Cortex	17.9^e^	2.76	Kastner et al. (2009)
C57Bl/6^d^	Female	Auto‐immune (anti‐mouse glomerular basement membrane serum)	Medulla	17.9^e^	1.18	Kastner et al. (2009)
C57Bl/6	Both^b^	Inducible glomerular podocin deletion	Cortex	>100^c^	2.13^f^	Xiao et al. (2021)
C57Bl/6	Male	Db/db	Whole	2.8	1.33	Veiras et al. (2021)
C57Bl/6	Female	Db/db	Whole	1.6	0.82	Veiras et al. (2021)

^a^ Albumin/creatinine.

^b^ Sexes not reported separately.

^c^ Protein/creatinine, roughly estimated from graph.

^d^ Likely background.

^e^ 24‐h protein, normalized to body weight.

^f^ Furin‐cleaved γ subunit fragment.

Resistance to doxorubicin‐induced proteinuria in 129S2/SvPas mice appears to occur because of their *Prkdc* genotype: Arg2140 in DNA‐PKcs, the same genotype as C57Bl/6 mice (Watanabe et al., 2021). Despite sharing the 129 background, 129S1/SvImJ mice exhibit a polymorphism (Arg2140Cys) in DNA‐PKcs that reduces DNA‐PKcs expression, enhancing sensitivity to doxorubicin (Watanabe et al., 2021). Published genotypes of in‐bred mouse strains are summarized in Table 2.

**TABLE 2:** *Prkdc* genotype in various mouse strains.

Mouse background	Substrain	Amino acid at position 2140	References
129Sv	129/SvJ	Cys	Mori et al. (2001) and Faiola et al. (2003)
	129S1/SvImJ	Cys	Papeta et al. (2010)
	129S2/SvPas	Arg	Present study
BALB/c	BALB/c	Cys	Watanabe et al. (2021) and Papeta et al. (2010)
	BALB/cHeA	Cys	Mori et al. (2001)
	BALB/cByJ	Cys	Yu et al. (2001) and Fabre et al. (2011)
	BALB/cAnN	Cys^a^	Mori et al. (2001)
C57Bl/6	C57Bl/6J	Arg	Papeta et al. (2010), Watanabe et al. (2021), and Mori et al. (2001)
	C57Bl/6 (Taconic)	Arg	Faiola et al. (2003)
	C57BL/6ByJ	Arg	Yu et al. (2001) and Fabre et al. (2011)
DBA/2N		Arg	Watanabe et al. (2024)
FVB/N		Arg	Mori et al. (2001)
STS/A		Arg	Mori et al. (2001)

^a^ Inferred based on restriction endonuclease analysis.

A challenging question is the explanation for increased resistance to doxorubicin‐induced proteinuria in female mice. Other studies have also reported enhanced susceptibility to proteinuria in male mice compared with females (Barsha et al., 2016; Ishola Jr et al., 2005; Veiras et al., 2021). Mechanisms underlying this difference remain to be identified.

## 5 CONCLUSION

In summary, 129S2/SvPas mice appear to exhibit reduced proteinuria when treated with doxorubicin, as compared to mice with the Arg2140Cys polymorphism in DNA‐PKcs. The low‐level proteinuria observed following doxorubicin treatment in these mice is not associated with proteolytic activation of ENaC, suggesting that this mouse background and doxorubicin dose may not be suitable for studies examining doxorubicin‐induced stimulation of ENaC proteolysis.

## FUNDING INFORMATION

This study was supported by an American Society of Nephrology Carl W. Gottschalk Research Scholar Grant (ECR), and grants from the National Institutes of Health grants R01DK139177 (ECR), K01DK140634, F32DK136356 (AJN), R01HL147818 (TRK), and U54DK137329 (TRK).

## CONFLICT OF INTEREST STATEMENT

Authors declare there are no conflicts of interest.

## ETHICS STATEMENT

All animal procedures were reviewed and approved by the University of Pittsburgh Institutional Animal Care and Use Committee (IACUC) and were conducted in accordance with the National Institutes of Health Guide for the Care and Use of Laboratory Animals and with the policies of the American Physiological Society, including adherence to the principles of replacement, reduction, and refinement.

## Supporting information


Appendix S1.

## Data Availability

All primary data is available in Appendix S1.
