# Supplementary material for: Resistance to doxorubicin‐induced proteinuria and proteolytic activation of ENaC in 129S2/SvPas mice
Source: Physiol Rep. 2025 Dec 8;13(23):e70667. doi: 10.14814/phy2.70667 (PMC12686598; doi:10.14814/phy2.70667)
Supplement: Supplementary file 1 — Appendix S1. [file PHY2-13-e70667-s001.pdf]

URINE ALBUMIN (Fig 1A)

mg/g

	Males	Males		Females	Females
Mouse	Pre-Doxorul	Post-Doxorubicin	Mouse	Pre-Doxorul	Post-Doxorubicin
1	1.02E+02	2.94E+02	1	2.14E+01	7.84E+02
2	5.03E+02	6.66E+03	2	2.60E+01	9.55E+02
3	1.43E+02	2.10E+03	3	2.50E+01	1.25E+02
4	8.55E+01	1.94E+03	4	1.43E+01	7.95E+01
5	7.53E+01	1.55E+03	5	1.86E+01	5.18E+01
6	2.57E+01	7.11E+02	6	1.54E+01	6.93E+01
7	1.05E+01	2.80E+03	7	1.18E+01	2.27E+02
			8	1.28E+01	7.72E+01
			9	9.03E+00	2.91E+02
			10	1.19E+01	1.30E+02

MOUSE BODY WEIGHTS (Fig 1B)

Weight (g)

	Mous 1	Mous 2	Mous 3	Mous 4	Mous 5	Mous 6	Mous 7	Mous 8	Mous 9	Mous 10	Mous 11	Mouse 12
**Day**												
**0**	26	26	20	29	25	26	27	25	25	23	27	25
**1**	26	26	21	29	24	26	26	24	24	23	27	25
**3**	25	25	18	28	23	24	25	23	22	21	25	22
**5**	24	23	18	28	23	24	25	24	22	21	25	22
**7**	25	22	19	28	24	25	25	23	22	21	25	22
**9**	26	23	19	27	24	25	26	23	22	21	25	23
**10**	26	24	19	28	25	25	27	24	23	21	25	23

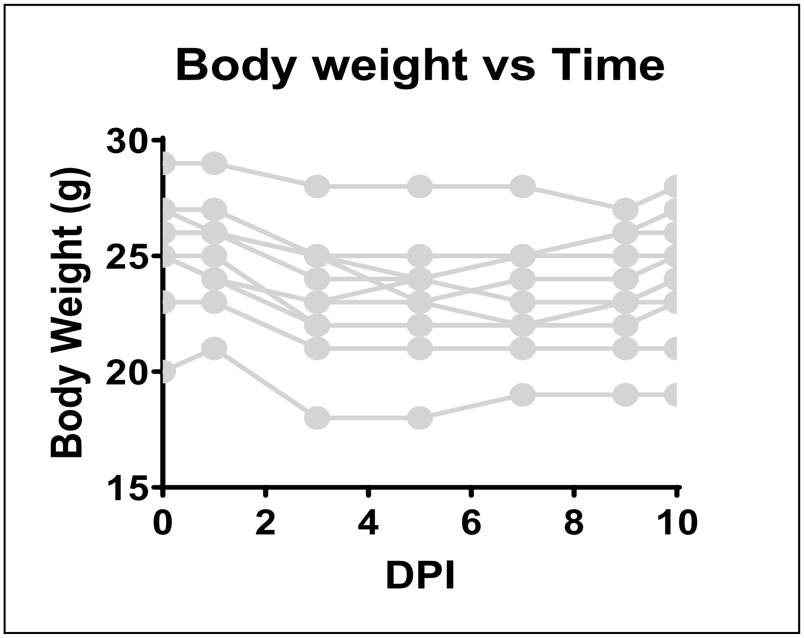

IMMUNOBLOT BAND QUANTIFICATION (Fig 2)

	Control Mice	Doxorubicin-treated mice
**Full-Length Gamma Subunit Abundance / Stain Free Gel**		
	1.25E+00	1.31E+00
	1.01E+00	1.51E+00
	8.56E-01	1.29E+00
	8.31E-01	1.04E+00
	1.06E+00	1.08E+00
		1.38E+00
**Distally-Cleaved Gamma Subunit Abundance / Stain Free Gel**		
	7.42E-01	1.28E+00
	9.40E-01	1.19E+00
	9.54E-01	1.09E+00
	1.34E+00	1.32E+00
	1.02E+00	1.17E+00
		1.05E+00
**Furin-Cleaved Gamma Subunit Abundance / Stain Free Gel**		
	9.68E-01	1.53E+00
	1.08E+00	1.64E+00
	1.01E+00	1.17E+00
	1.16E+00	1.68E+00
	7.83E-01	1.59E+00
		1.41E+00
**Distally-Cleaved Gamma Subunit / Total Gamma Subunit**		
	1.21E-01	1.73E-01
	1.69E-01	1.45E-01
	1.92E-01	1.59E-01
	2.48E-01	1.99E-01
	1.84E-01	1.80E-01
		1.44E-01
**Furin-Cleaved Gamma Subunit / Total Gamma Subunit**		
	1.37E-01	1.80E-01
	1.69E-01	1.76E-01
	1.78E-01	1.49E-01
	1.88E-01	2.23E-01
	1.22E-01	2.13E-01
		1.69E-01

BLOOD DATA (Fig 3)

	Control Mice	Doxorubicin-treated mice
**Blood Na (mmol/L)**	145	148
	144	148
	145	148
	144	144
	144	146
		144
**Blood K (mmol/L)**	4.5	4.3
	4.6	4.9
	4.8	3.6
	4.4	5.2
	4	4.2
		4.4
**Blood Cl (mmol/L)**	110	115
	111	111
	116	118
	114	115
	110	117
		112
**Blood tCO2 (mmol/L)**	23	22
	22	24
	18	19
	20	19
	23	21
		22
**BUN (mg/dL)**	30	28
	24	27
	32	32
	22	20
	24	26
		20
**Hemoglobin (mg/dL)**	13.3	12.9
	14.6	13.3
	13.9	13.3
	14.6	13.6
	15.3	12.9
		14.3

URINE PARAMETERS AFTER BENZAMIL TREATMENT (Fig 4)

	**Untreated**	**After Doxorubicin**
**Urine Albumin in Benzamil-Treated Animals**	1.04E+01	1.16E+02
mg/hr	6.24E+00	2.30E+02
	1.28E+01	6.31E+00
	9.79E+00	4.53E+02
	7.40E+00	2.02E+02
	6.44E+00	3.25E+01
**UNaV in Benzamil-Treated Animals**	1.82E-02	2.92E-02
micromol/hr	1.04E-02	1.54E-02
	2.23E-02	8.80E-03
	2.10E-02	1.82E-02
	1.80E-02	1.15E-02
	1.64E-02	8.51E-03
**UKV in Benzamil-Treated Animals**	4.70E-03	6.06E-03
micromol/hr	5.68E-03	5.99E-03
	6.50E-03	2.34E-03
	1.54E-02	1.14E-02
	1.32E-02	1.34E-02
	6.37E-03	1.86E-03
**UClV in Benzamil-Treated Animals**	1.16E-02	1.99E-02
micromol/hr	8.46E-03	9.21E-03
	1.64E-02	7.44E-03
	1.58E-02	1.30E-02
	1.43E-02	1.02E-02
	1.27E-02	4.69E-03
**Urine Na/Urine K in Benzamil-Treated Anim**	3.88E+00	4.83E+00
(no units)	1.83E+00	2.57E+00
	3.43E+00	3.76E+00
	1.36E+00	1.59E+00
	1.37E+00	8.63E-01
	2.58E+00	4.57E+00
**Urine Volume in Benzamil-Treated Animals**	6.50E-01	8.10E-01
mL	3.70E-01	4.60E-01
	8.00E-01	3.70E-01
	5.10E-01	5.90E-01
	4.10E-01	5.90E-01
	3.60E-01	3.30E-01
